# Midline crossing pulmonary vein: right upper lobe dual venous drainage, with partial anomalous venous return of the right lung into a persistent left superior vena cava

**DOI:** 10.1007/s00276-021-02849-9

**Published:** 2021-10-28

**Authors:** J. van Schuppen, A. E. van der Hulst, I. M. Kuipers, B. Straver, S. M. Boekholdt, R. N. Planken, R. J. Oostra

**Affiliations:** 1 Department of Radiology and Nuclear Medicine, Amsterdam University Medical Center, Location AMC, Meibergdreef 9, Suite C1-234, 1105 AZ Amsterdam, The Netherlands; 2 Department of Pediatric Cardiology, Amsterdam University Medical Center, Location Meibergdreef, Amsterdam, The Netherlands; 3 Department of Cardiology, Amsterdam University Medical Center, Location Meibergdreef, Amsterdam, The Netherlands; 4 Department of Medical Biology, Section Clinical Anatomy and Embryology, Amsterdam University Medical Center, Location Meibergdreef, Amsterdam, The Netherlands

**Keywords:** Pulmonary veins, Persistent left superior vena cava, Congenital abnormalities, Multimodal imaging, Computed tomography angiography

## Abstract

**Introduction:**

We present a case of dual drainage of the right upper lobe of the lung into the left atrium and via partial anomalous venous pulmonary return (PAPVR) into a persistent left superior vena cava (SVC).

**Discussion:**

It is only in the minority of PAPVR cases where the anomalous pulmonary veins cross the midline. We provide a review of current literature on this topic and an explanatory embryological model. Knowledge of embryonic development and possible anatomic variations, including the concept of dual venous drainage of the lung, leads to better interpretation of imaging, with more accurate description of the morphology at hand. High-resolution multidetector computed tomography (MDCT) helps to delineate the exact vascular anatomy. This will enhance a better understanding of and anticipation on the patient’s disease status, with more accurate planning of intervention, and possibly less complications.

## Case presentation

**Fig. 1:**
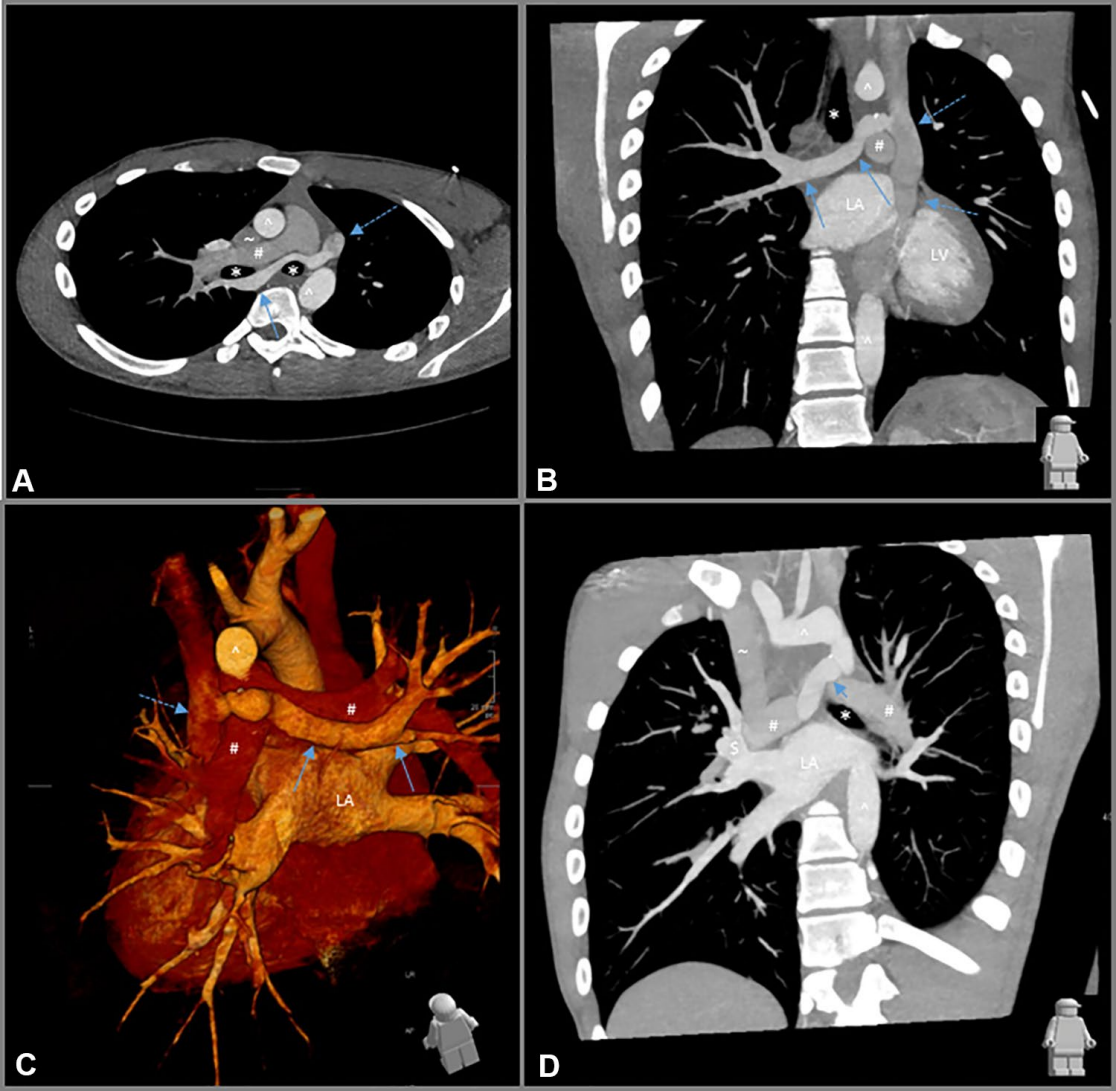
ECG triggered Turboflash CTA of the chest. Oblique axial reformat at the level of the anterior mediastinum, demonstrating the anomalous right upper lobe vein (blue arrow) traversing behind the right main bronchus (*), right pulmonary artery (#), the left main bronchus (*) anterior of the descending aorta (^), draining into the persistent left superior vena cava (SVC) (dotted arrow). Right SVC (~). **B** Coronal oblique reformat demonstrating the course of the anomalous right upper lobe vein (blue arrow) into the persistent left SVC (dotted arrow), *LA* left atrium, *LV* left ventricle. Aortic arch (^). Left pulmonary artery (#). Trachea (*). **C** 3D rendering looking from the backside at the heart. *LA* left atrium. Aortic arch (^). **D** Coronal oblique thick slab reformat, showing the normal venous drainage ($) to the left atrium (LA). Left bronchus (*). Pulmonary arteries (#). Right SVC (~). Anomalous right upper lobe vein (blue arrow). Aortic arch and descending aorta (^). Anomalous right upper lobe vein (blue arrow)

We present a 17-year-old boy with a coincidental finding of partial anomalous pulmonary venous return (PAPVR). At 3 years of age, a murmur was noticed during routine physical exam. Echocardiography revealed an atrial septum defect (ASD) type II, an asymmetrical aortic valve with mild stenosis, as well as a persistent left superior vena cava (SVC) draining into the coronary sinus. In addition, it was suspected on echocardiography that one of the pulmonary veins drained into the persistent left SVC. Cardiac catheterization confirmed this, showing an abnormal connection of the right upper pulmonary vein into the persistent left SVC. As the left-to-right shunt was clinically acceptable and there were no symptoms, the patient was followed up without intervention. During follow-up, no clinical complaints occurred. He reported no palpitations and a normal exercise tolerance. The resting ECG showed normal sinus rhythm, left axis deviation and a right bundle branch block. At the age of 17, cardiac MRI showed right ventricular dilatation (EDV/BSA 144 ml/m^2^) and a Qp/Qs ratio of 1.2. Subsequently, an ECG triggered Turboflash CTA of the chest (Somatom Force, Siemens Healthineers, Erlangen, Germany), using a tube current of 80 kV was performed to further determine the exact anatomy and possibilities for intervention (Fig. 1). A dual pulmonary venous return of the right upper lobe into the persistent left SVC and into the left atrium was observed. The anomalous pulmonary vein showed a remarkable course crossing the midline behind the right main bronchus, and anterior of the left bronchus into the left SVC. The coronary sinus was normally developed. The right lung had three normally developed lobes.

## Discussion

In pulmonary venous development, there is a broad spectrum of variants and anomalies known, ranging from supernumeraries and abnormal diameter of veins to variants in drainage to the left atrium. In addition, anomalous veins draining in the systemic circulation and abnormal connections with pulmonary arteries are known congenital variants [6, 8].

**Fig. 2:**
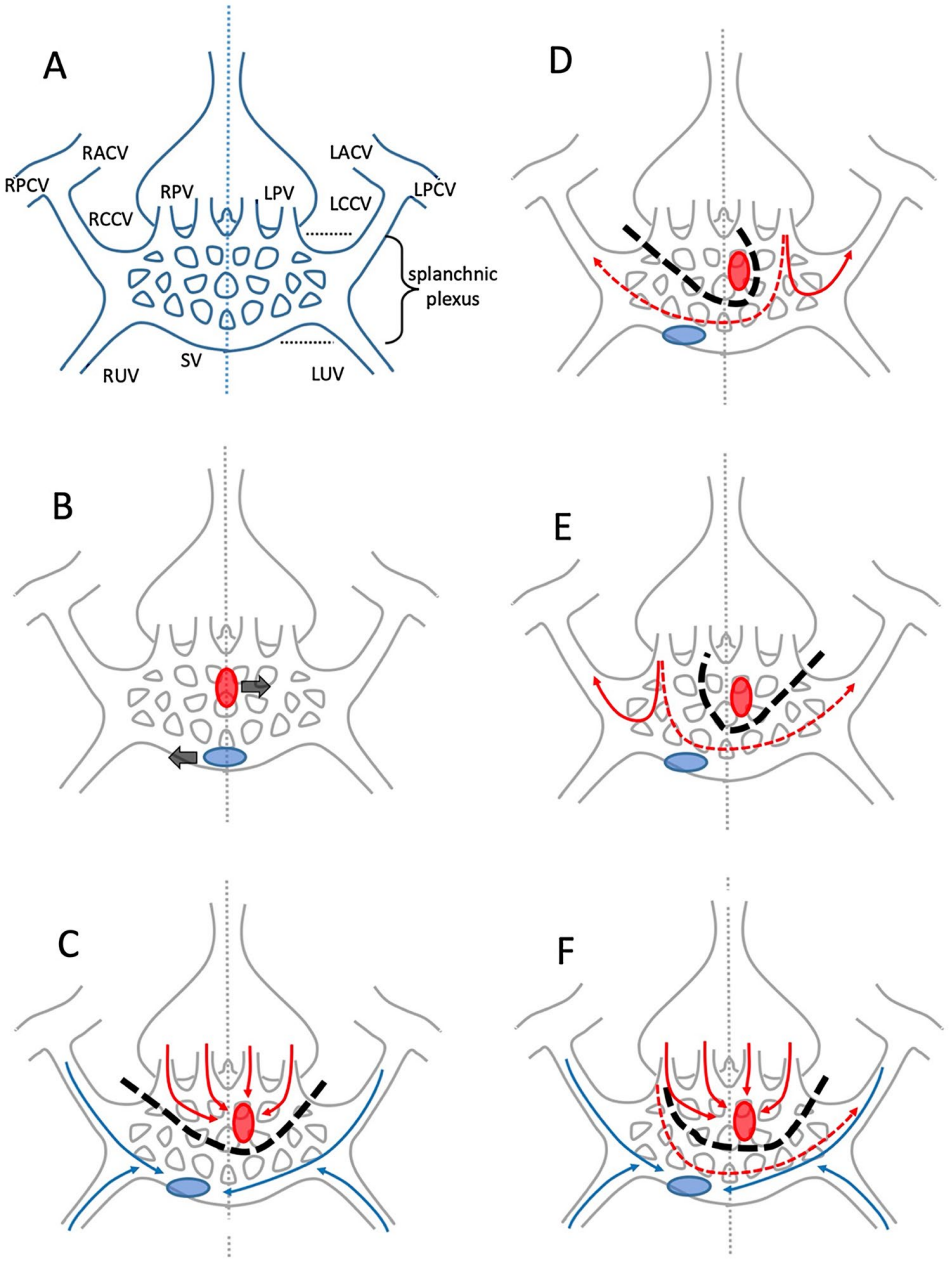
**A** Schematic representation of the developing venous pole (modified after Lyen et al. [6]), where the systemic, umbilical and vitelline circulations of the embryo drain (see list of abbreviations). For the sake of simplicity, the heart (positioned in front of the venous pole), the azygos system and most of the abdominal part of the splanchnic plexus have not been depicted in these figures. **B** Locations of the systemic inflow of the heart [blue ellipse] and of the atrial protrusion connecting with the (pulmonary part of the) splanchnic plexus [red ellipse], which move to the right and left sides of the midline [dotted vertical line], respectively. **C** Division of the splanchnic plexus [dashed black line], with selective drainage of its superior most part on the left (side of the) atrium [red arrows] and the remainder of the plexus, together with the cardinal and umbilical systems [blue arrows], to the right (side of the) atrium. **D**, **E** Abnormalities in the drainage of pulmonary veins [red arrows] resulting from aberrations in the process of proliferation and regression of the splanchnic plexus contributions, including midline crossing veins [dashed red arrows]. **F** In this variant, only part of the splanchnic plexus divides [dashed black line], which results in dual drainage of part of the lung on the left (side of the) atrium [red arrows] as well as drainage on the right (side of the) atrium [dashed red arrows]. See text for further explanation

During fetal development of the venous pole, three distinct circulatory systems drain, directly or indirectly, on a collective vessel known as the sinus venous, before entering the heart (Fig. 2A): (1) the somatic circulation of the embryo proper, consisting of the right and left anterior and posterior cardinal veins that drain on the common cardinal veins (CCV); (2) the right and left umbilical veins that return oxygenated blood from the placenta; (3) the vitelline circulation that gives rise to the splanchnic plexus, which drains the internal organs, including the developing lungs by means of the right and left pulmonary veins.

During subsequent development, the initial left–right symmetry is disrupted as soon as the left-sided cardinal and umbilical systems start to regress and the systemic inflow of the heart moves to the right as a result of differential growth (Fig. 2B). Meanwhile, the embryonic atrial lumen forms a protrusion that connects with the (pulmonary part of the) splanchnic plexus; this protrusion will become confined to the left side of the atrium when septation commences [2]. As a result of selective proliferation and regression, the mesh of vessels in the splanchnic plexus becomes divided in such a way that its superior most part, including the pulmonary veins, will exclusively drain on the left (side of the) atrium; whereas the drainage of the remainder of the plexus, together with the cardinal and umbilical systems, becomes confined to the right (side of the) atrium (Fig. 2C). At the end of the embryonic period, the right CCV has become the superior caval vein, the left CCV has either regressed and transformed into the coronary sinus or, rarely may persist as a left-sided superior caval vein, and the splanchnic vessels have formed the inferior caval vein [2, 8].

Aberrations in the process of proliferation and regression of the splanchnic plexus contributions can lead to various abnormalities in the drainage of pulmonary veins, mostly to adjacent ipsilateral but also to contralateral systemic veins (Fig. 2D, E). This not only concerns anomalies in the division of the plexus in a pulmonary and an abdominal part, but also the occasional persistence of vessels that may cross the midline, resulting, e.g., in drainage of a left pulmonary vein (LPV) on (tributaries of) the superior or inferior vena cava or of a right pulmonary vein (RPV) draining on a coronary sinus or a persisting left SVC. In its mildest configuration, the anomalous vessel drains only a confined part of the lung, resulting in dual drainage of the affected lobe to both the left atrium as well as to a systemic vein as in the case described here (Fig. 2F).

Prevalence of PAPVR is reported between 0.4 and 0.7%. The most common variant in PAPVR is a right-sided partial return to the right-sided SVC at the level where the SVC drains into the right atrium, but may occur in any systemic vein or right atrium. Right-sided variants of superior vein drainage are associated with a superior sinus venosus ASD. Left-sided PAPVR occurs in 18%. The most common form of left-sided PAPVR is drainage of left upper pulmonary veins in the left brachiocephalic vein. This may mimic a persistent left SVC on CT. Other variants are drainage in the hemi-azygos vein, persistent left SVC, coronary sinus, inferior vena cava or portal veins [2, 6–8]. It is only in the minority of PAPVR cases where the anomalous pulmonary veins cross the midline [9].

A left-sided SVC is seen in 0.3–0.5% of the normal population, with a higher occurrence in congenital heart disease. This results from failure of regression of the left anterior cardinal vein. There is an association with congenital cardiac abnormalities as PAPVR, septal defects, and coarctation of the aorta [10].

Imaging is used to create an overview of anatomy and congenital abnormalities, and aides in planning interventions. In imaging of PAPVR, often the first modality is ultrasound, although abnormalities in pulmonary veins can be challenging to visualize due to limited views. Chest X-ray may give a hint of abnormal anatomy, e.g., widened mediastinum, but is not of added value in analysis of PAPVR. Intravenous contrast-enhanced multidetector computed tomogram (MDCT) with thin slices provides an excellent depiction of cardiac anatomy and vasculature, creating a good overview of normal and abnormal pulmonary venous connections. Due to rapid imaging possibilities in up to date generation scanners, MDCT can often be performed without sedation. In addition, ECG-gated protocols create scans without cardiac movement, with high spatial resolution in isotropic voxels. Furthermore, possibilities of 3D reconstruction help visualize abnormalities. In addition, MRI may be used to evaluate venous structures and drainage, which is an important tool to quantify cardiac function and the size of left-to-right shunt. Lack of radiation is an important advantage of MRI compared to CT, although scanning time and the potential need for anesthetic in young children are a disadvantage [3, 6].

A PAPVR creates a left-to-right shunt. Younger patients are usually asymptomatic, but also may present with non-specific complaints, or complaints of exercise intolerance. Often PAPVRs are coincidental findings later in life. A significant left-to-right shunt results in volume overload of the right ventricle. This leads to dilatation of right atrium and ventricle with eventually right heart failure, and may also result in pulmonary hypertension. Early detection and treatment of this entity can prevent evolution to right-sided heart failure and pulmonary hypertension in younger patients. Indication for treatment is a shunt with a pulmonary to systemic flow ratio (Qp:Qs) larger than 1.5, or a right ventricle dilatation with an end diastolic volume of more than 150 ml/m^2^ [1].

This case is a rare configuration of dual drainage of a right upper lobe, combined with a PAPVR that crosses the midline and drains in a persistent left SVC. The closest resemblance of this unusual PAPVR would be drainage of a RPV into the coronary sinus [4]. Left pulmonary veins crossing the midline and draining in right systemic veins have been described previously [5, 9].

Knowledge of embryonic development and possible anatomic variations, including the concept of dual venous drainage of the lung, leads to better interpretation of imaging, with more accurate description of the morphology at hand. High-resolution MDCT helps to delineate the exact vascular anatomy. This will enhance a better understanding of and anticipation on the patient’s disease status, with more accurate planning of intervention, and possibly less complications. In our case, due to the presence of dual venous return, percutaneous closure of the PAPVR was possible, instead of correction via open heart surgery.

## Data Availability

Not applicable.

## Abbreviations

ASD: Atrial septum defect
BSA: Body surface area
CTA: Computed tomography angiography
EDV: End diastolic volume
LACV: Left anterior cardinal vein
LCCV: Left common cardinal vein
LPCV: Left posterior cardinal vein
LPV: Left pulmonary vein
LUV: Left umbilical vein
MDCT: Multidetector computed tomography
MRI: Magnetic resonance imaging
PAPVR: Partial abnormal pulmonary venous return
Qp: Pulmonary flow
Qs: Systemic flow
RACV: Right anterior cardinal vein
RCCV: Right common cardinal vein
RPCV: Right posterior cardinal vein
RPV: Right pulmonary vein
RUV: Right umbilical vein
SV: Sinus venosus
SVC: Superior vena cava
